# The timing of azithromycin treatment is not associated with the clinical prognosis of childhood *Mycoplasma pneumoniae* pneumonia in high macrolide-resistant prevalence settings

**DOI:** 10.1371/journal.pone.0191951

**Published:** 2018-01-29

**Authors:** Dehua Yang, Linghong Chen, Zhimin Chen

**Affiliations:** 1 Zhejiang University School of Medicine, Hangzhou, Zhejiang, P.R. China; 2 The First People’s Hospital of Wenling, Wenling, Zhejiang, P.R. China; University of Kentucky, UNITED STATES

## Abstract

**Background:**

*Mycoplasma pneumoniae* infection is a major cause of community-acquired pneumonia in children. We performed a retrospective study to evaluate the clinical impact of the timing of azithromycin treatment in children with *Mycoplasma pneumoniae* pneumonia in high macrolide-resistant prevalence settings.

**Methods and findings:**

A total of 623 patients were enrolled in this study and were divided into 2 groups according to the timing of azithromycin therapy. Children who received azithromycin within 3 days (72 hours) after the onset of *Mycoplasma pneumoniae* pneumonia were classified into the early azithromycin treatment group (n = 174), whereas the late azithromycin treatment group (n = 449) comprised children treated with azithromycin more than 72 hours after symptom onset. We evaluated clinical prognosis according to demographic, clinical and laboratory characteristics. Although the early azithromycin treatment group exhibited a longer fever duration after azithromycin administration (7.17±4.12 versus 4.82±3.99 days, P<0.01), the total fever duration exhibited no significant difference (9.02±4.58 versus 9.57±4.91 days, P = 0.212). After azithromycin therapy, the two groups exhibited no significant differences with respect to improvements in the laboratory and radiological findings (all P>0.05).

**Conclusion:**

The timing of azithromycin treatment is not associated with the clinical prognosis of *Mycoplasma pneumoniae* pneumonia in children in high macrolide-resistant *Mycoplasma pneumoniae* prevalence settings.

## Introduction

*Mycoplasma pneumoniae* (*M*. *pneumoniae*) pneumonia is a common disease in children, representing 10–30% of all pediatric cases of community-acquired pneumonia[1–2]. Macrolides are suitable for children with *M*. *pneumoniae* pneumonia, and azithromycin remains the first-choice treatment due to its well tolerance and compliance. Azithromycin has been believed to exhibit satisfactory effects in the treatment of *M*. *pneumoniae* pneumonia in the past[3–6]. However, there have been an increasing number of cases of *M*. *pneumoniae* pneumonia as well as increasing severity in recent years[7–10]. Emergence of macrolide-resistant *Mycoplasma pneumoniae* (MRMP), which is much more severe in eastern Asia[8–10], may be one of the most important reasons. However, there is no enough evidence proving that macrolides are invalid in treating MRMP in vivo. We can not design a research of treating children infected MRMP without macrolides as ethics violation. Therefore, we made a retrospective research and compared outcomes of different timing of azithromycin administration to reflect its effect in high macrolide-resistant prevalence settings.

## Methods

### Ethics statement

The Ethics Committee of the Children’s Hospital, Zhejiang University School of Medicine, approved the study. Data were accessed anonymously and used as statistical analysis only, exempting us from informed consent.

### Subjects

The medical records of children admitted to the Children’s Hospital, Zhejiang University School of Medicine in China from January 2011 to December 2014 were collected. Children with the criteria below were enrolled:

1. Pneumonia: fever (>37.5°C), cough, abnormal breath sounds on auscultation and abnormal chest X-rays[11].
2. Positive *M*. *pneumoniae* polymerase-chain-reaction (PCR) testing of nasopharyngeal secretions or bronchoalveolar lavage fluid.
3. Severe *M*. *pneumoniae* pneumonia: The disease severity was defined by scores from 0 to 5 based on the following clinical findings according to a previous studies with modification: rapid breathing or use of accessory muscles of respiration, fever (>38.5°C), more than two affected pulmonary lobes on chest X-rays, and hospital stay greater than seven days. Severity scores of 3 or greater were considered severe pneumonia[12–13].

### Exclusion criteria

Patients were excluded if they had chronic cardiac or pulmonary diseases, immunodeficiency or infections with other pathogens detected during treatment.

### Study design

This was a retrospective study. All of the enrolled subjects received sequential azithromycin therapy (10 mg/kg/day for 3–5 days, withdraw for 3–4 days, and then 10 mg/kg/day for 3 days). These patients were classified into two groups according to the timing of azithromycin therapy. Patients who received azithromycin within 3 days (72 hours) after the onset of *M*. *pneumoniae* pneumonia were classified into the early administration group, whereas the late administration group comprised children treated with azithromycin more than 3 days (72 hours) after symptom onset.

### Data collection

The following data were recorded: age, sex, duration of fever, chest radiographic findings, severity scores, extrapulmonary complications, and blood tests, including white blood cell (WBC) count, neutrophil count, and C-reactive protein (CRP).

### Statistical analysis

SPSS (Version 18; SPSS, Chicago, IL, USA) was used to analyze the data. Normally distributed data are expressed as the mean±SD. An independent sample t test was utilized to compare the data. Data with a skewed distribution were expressed as median values (25th-75th interquartile ranges). The comparisons were made by Mann-Whitney U test. Chi-square tests were used to compare the categorical data. The statistical significance was defined as P≤0.05.

## Results

### Basic clinical data

Six hundred twenty-three patients ranged from 0.30 to 15.92 years were enrolled. One hundred seventy-four patients were classified in the early group (98 males, 76 females), with a median age of 4.54 (2.65~7.19) years. In this group, the median WBC count before azithromycin therapy was 7.52 (6.20~10.09)×10^9^/L with (59.28±16.07)% neutrophils, and the CRP was 10.15 (5.00~21.00) mg/L. Four hundred forty-nine patients were enrolled in the late group (243 males, 206 females), with a median age of 3.75 (2.08~6.67) years. In this group, the median WBC count before azithromycin therapy was 8.04 (6.50~10.10)×10^9^/L with (57.83±16.48)% neutrophils, and the CRP was 10.00 (4.00~24.00) mg/L. The group characteristics, including sex, age, WBC, neutrophils, and CRP did not differ significantly (Table 1).

**Table 1:** Clinical characteristic of the early and late administration groups.

Clinical information	Early administration (n = 174)	Late administration (n = 449)	t/χ2-value	P-value
Sex, male/female	98/76	243/206	0.245	0.620
Age, years	4.54(2.65~7.19)	3.75(2.08~6.67)	1.948	0.051
WBC, ×10^9^/L	7.52(6.20~10.09)	8.04(6.50~10.10)	1.252	0.210
Neutrophils, %	59.28±16.07	57.83±16.48	0.993	0.321
CRP, mg/L	10.15(5.00~21.00)	10.00(4.00~24.00)	0.366	0.714
Fever duration before azithromycin therapy, days	1.84±1.08	4.75±2.47	19.546	<0.001
Fever duration after azithromycin therapy, days	7.17±4.12	4.82±3.99	6.352	<0.001
Total fever duration, days	9.02±4.58	9.57±4.91	1.251	0.212

There was no significant difference in the total fever duration between these two groups (P = 0.212). Fever duration before azithromycin administration in the early group was significantly shorter than that of the late group (1.84±1.08 versus 4.75±2.47 days, t = 19.546, P<0.01). Fever duration after azithromycin administration was 7.17±4.12 days for the early administration group and 4.82±3.99 days for the late administration group, which represented a statistically significant difference (P<0.001; Table 1).

### Severity of *M*. *pneumoniae* pneumonia

We also evaluated the severity of *M*. *pneumoniae* pneumonia, including the presence of severe pneumonia, pulmonary atelectasis, and pleural effusion, and detected no significant differences between these two groups (P = 0.442, 0.499, 0.301, respectively). However, extra-pulmonary complications were less common in the early administration group compared with the late administration group (χ2 = 5.127, P = 0.024), as shown in Table 2.

**Table 2:** Pneumonia severity in the early and late administration groups.

Radiological features	Early administration (n = 174)	Late administration (n = 449)	χ2-value	P-value
Severe pneumonia, %	19(10.92%)	40(8.91%)	0.591	0.442
Pulmonary atelectasis, %	31(17.82%)	70(15.59%)	0.457	0.499
Pleural effusion, %	34(19.54%)	105(23.39%)	1.070	0.301
Extra-pulmonary complications, %	38(21.84%)	139(30.96%)	5.127	0.024

Data are presented as numbers (percentages).

### Therapeutic effects

The WBC and CRP of most patients recovered within 10 days after azithromycin therapy. Atelectasis and pleural effusion did not resolve until one month post-treatment. The improvements in the laboratory and radiological findings did not differ significantly between the early and late administration groups (all P>0.05; Table 3).

**Table 3:** Clinical efficacy of azithromycin therapy.

Clinical efficacy	Early administration (n = 174)	Late administration (n = 449)	χ2-value	P-value
Routine blood test recovery rate, %				
WBC	93.75%(15/16)	95.45%(42/44)	0.069	0.793
CRP	97.01%(65/67)	97.78%(176/180)	0.000	1.000
Atelectasis resolution, %				
Two weeks later	12.90%(4/31)	14.29%(10/70)	0.000	1.000
One month later	22.58%(7/31)	35.71%(25/70)	1.712	0.191
Pleural effusion resolution, %				
Two weeks later	35.29%(12/34)	32.38%(34/105)	0.098	0.754
One month later	58.82%(20/34)	57.14%(60/105)	0.073	0.788

Data are presented as percentages (recovery number/total abnormal number).

## Discussion

In our study, there was no significant difference between the early group and the late group with respect to total fever duration, nor were there any differences with respect to severity or therapeutic effects. According to these results, the early use of azithromycin did not shorten the fever duration or contribute to recovery, which means azithromycin therapy may be ineffective. A third group without azithromycin treatment as a reference would be more convincing, but due to the ethical issues surrounding this protocol, there remains no further proof.

Biondi et al[14] reported there was insufficient evidence to support the efficacy of macrolides in community-acquired lower respiratory tract infection of *M*. *pneumoniae* in children. Macrolide-unresponsive *M*. *pneumoniae* pneumonia is believed to represent infection with macrolide-resistant strains[15–17] and/or to represent an overactivation of the immune response[18–19]. Transition mutations at A2063G in domain V of the 23S rRNA gene are present in MRMP strains, and these mutations induce high-level resistance to macrolides with minimum inhibitory concentration (MIC) of 8 to >256 μg/ml[15–17]. In our study, the total fever duration of 9.41±4.82 days was much longer than that of macrolide-sensitive *M*. *pneumoniae* (MSMP) infections[15–16] and early reports[6] but was similar to that of MRMP infections[15–16]. Cases of MRMP are increasing annually, especially in Asia. For example, in Japan, Morozumi et al. have reported that the prevalence of MRMP increased rapidly from 5.0% in 2003[20] to 39% in 2008[9], and Okada et al. reported rates of 87.1% in 2011[10]. In our country, MRMP represented 83%[21], 87.7%[22], and 92%[23] of *M*. *pneumoniae* pneumonia in Shanghai, Hangzhou, and Beijing, respectively, which is significantly higher than the rates in Europe and the United States[7, 24–26]. These findings suggest that the cases of *M*. *pneumoniae* in our study may be MRMP.

An over-reactive immune response is always presents in *M*. *pneumoniae* infection, especially in cases with extra-pulmonary complications. Azithromycin is reported to possess immune regulation effects, which are believed to be beneficial in cases of *M*. *pneumoniae* pneumonia[27–30]. In our study, 28.41% of cases exhibited extra-pulmonary complications, including myocardial damage, liver function abnormalities, and dermatologic damage. The early administration group exhibited significant fewer extra-pulmonary complications than did the late administration group, which suggests that the early use of azithromycin may help reduce extra-pulmonary complications.

We were unable to isolate MRMP strains from our patients as it was a retrospective study. It would be more convincing if we confirmed patients infected with MRMP though the incidence of MRMP was more than 80% in our city[22].

Our retrospective study provided evidence to support the conclusion that the timing of azithromycin treatment is not associated with the clinical prognosis of *M*. *pneumoniae* pneumonia in children in high macrolide-resistant prevalence settings, with the exception of extra-pulmonary complications. Azithromycin may be ineffective in treating MRMP pneumonia in vivo, but we are unable to withhold antibiotic treatment to confirm test this hypothesis due to ethical issues. However, the early use of azithromycin aids in reducing extra-pulmonary complications in *M*. *pneumoniae* pneumonia.
